# Congenital Lactase Deficiency: Mutations, Functional and Biochemical Implications, and Future Perspectives

**DOI:** 10.3390/nu11020461

**Published:** 2019-02-22

**Authors:** Dalanda Wanes, Diab M. Husein, Hassan Y. Naim

**Affiliations:** 1 Department of Physiological Chemistry, University of Veterinary Medicine Hannover, Bünteweg 17, 30559 Hannover, Germany; dalanda.wanes@tiho-hannover.de (D.W.); diab.husein@tiho-hannover.de (D.M.H.); 2 Laboratory of Functional Physiology and Valorization of Bioresources–Higher Institute of Biotechnology of Béja, University of Jendouba, Jendouba 8189, Tunisia

**Keywords:** carbohydrate malabsorption, lactase-phlorizin hydrolase, congenital lactase deficiency, compound heterozygote inheritance

## Abstract

Congenital lactase deficiency (CLD) is a severe autosomal recessive genetic disorder that affects the functional capacity of the intestinal protein lactase-phlorizin hydrolase (LPH). This disorder is diagnosed already during the first few days of the newborn’s life due to the inability to digest lactose, the main carbohydrate in mammalian milk. The symptoms are similar to those in other carbohydrate malabsorption disorders, such as congenital sucrase-isomaltase deficiency, and include severe osmotic watery diarrhea. CLD is associated with mutations in the translated region of the LPH gene that elicit loss-of-function of LPH. The mutations occur in a homozygote or compound heterozygote pattern of inheritance and comprise missense mutations as well as mutations that lead to complete or partial truncations of crucial domains in LPH, such as those linked to the folding and transport-competence of LPH and to the catalytic domains. Nevertheless, the identification of the mutations in CLD is not paralleled by detailed genotype/protein phenotype analyses that would help unravel potential pathomechanisms underlying this severe disease. Here, we review the current knowledge of CLD mutations and discuss their potential impact on the structural and biosynthetic features of LPH. We also address the question of whether heterozygote carriers can be symptomatic for CLD and whether genetic testing is needed in view of the severity of the disease.

## 1. Introduction

Carbohydrates, also sugars and starches, constitute one main source of energy in our daily diet. Depending on their structural complexity, these compounds are categorized under monosaccharide, disaccharide, oligosaccharide or polysaccharide groups [1,2]. Carbohydrates are absorbed in the intestine only in the monosaccharide form; therefore, all of the higher structures need to be hydrolyzed to the simple sugars prior to absorption. Digestion of carbohydrates to disaccharides commences by the action of amylase in the saliva and pancreas and is accomplished in the intestine by a group of enzymes, the disaccharidases, which are located in the intestinal brush border membrane. These enzymes digest the disaccharides to monosaccharides, such as glucose, fructose, and galactose, which are then ultimately absorbed and transported into the cell interior via specific transporters, such as the Na(+)-dependent glucose transporter, SGLT1, that is located at the apical membrane of the enterocytes [3]. Prominent members of the intestinal enzymes that are implicated in the carbohydrate digestion are sucrase-isomaltase (SI), maltase-glucoamylase (MGA), and lactase-phlorizin hydrolase (LPH) [4]. SI and MGA hydrolyze α-glycosidically linked disaccharides which are ingested in the food or produced by the action of pancreatic amylase on the more complex sugars, such as starch [5]. LPH is the only β-galactosidase of the intestine that hydrolyzes the principle sugar in the mammalian milk, lactose, as well as some natural β-glycosides [6,7]. This enzyme has a crucial role during the infant period when milk is the exclusive source of nutrition [8,9].

## 2. Carbohydrate Malabsorption

The failure to digest disaccharides to monosaccharides due to enzyme deficiency results in carbohydrate malabsorption that causes oftentimes watery acidic diarrhea [10]. Osmotic diarrhea arises when nutrients (especially those of smaller size) cause an osmotic force, which drives water into the gut lumen [11]. Osmotic diarrhea regresses with discontinuation of oral feeding.

SI is associated with several malabsorption disorders, such as congenital sucrase-isomaltase deficiency (CSID) [12], irritable bowel syndrome (IBS) [13] or secondary malabsorption in inflammatory bowel diseases (IBD) [14] or intestinal infections [15].

Lactose intolerance is another intestinal digestion disorder that is linked to reduced or complete loss of LPH activity. Two major types of lactose intolerance exist, primary lactase deficiency, also called adult-type hypolactasia, and congenital lactase deficiency (CLD) [10]. In most mammals, the lactase activity is maximum at birth and during lactation, when milk is the exclusive nutrient. Thereafter, between weaning and before adulthood, the lactase activity decreases dramatically to 5–10% of the level at birth due to the absence of a lactase persistent allele [16]. By contrast, congenital lactase deficiency, a rare autosomal recessive inherited disease, is characterized by reduced or complete loss of lactase activity already at birth [17]. Finally, lactose malabsorption can be also induced by an injury of the small intestine, for example, from acute gastroenteritis, chemotherapy or intestinal parasites [18].

## 3. Congenital Lactose Intolerance

Unlike in several forms of genetically-determined or acquired forms of carbohydrate malabsorption disorders, congenital lactase deficiency is suspected in neonates of a few days of age with the onset of watery diarrhea after the start of breastfeeding, usually in the absence of vomiting, with adequate intake and no food refusal [19,20]. If the diagnosis is delayed, dehydration and metabolic acidosis can become severe and life-threatening. Early recognition of lactose intolerance is therefore crucial to avoid potential risks of dehydration of the newborn [21]. A prerequisite to achieving this fundamental task is a thorough knowledge of the cell and molecular biology and the corresponding genetics of lactase-phlorizin hydrolase. An essential criterion is the identification of the genetic inheritance pattern, homozygote or compound heterozygotes, the putative mutations in the LCT gene, their location within the multidomain structured LPH protein and ultimately their impact on the folding, trafficking, and function of LPH. Given that LPH is a dimeric protein [22], it is also important to address the crucial question of whether a mutant LPH impacts the wild type protein in heterozygote carriers.

## 4. Structural and Biosynthetic Features of LPH

LPH is composed of four homologous structural domains, which together cooperate to ensure a competent trafficking and efficient polarized sorting of the protein to the apical membrane [23]. Recent work has unraveled the role of each distinct domain in the intramolecular organization, biosynthesis, trafficking, and function of LPH. Domain I and part of domain II comprise the profragment of the precursor form of LPH, pro-LPH, which is an essential component in the acquisition of pro-LPH to its proper folding and subsequent function [24]. An interesting structural feature in LPH is a small polypeptide, L735-R868, that exerts a crucial role in modulating the trafficking behavior of LPH and its biological function. In fact, the association of this domain with other LPH domains that are transport-competent leads to their intracellular block in the ER. The unique *N*-glycosylation site in this domain is the site for its association with the lectin chaperone calnexin, thus facilitating the folding of the entire LPH molecule [25]. Domain III comprises the phlorizin hydrolase active site that possesses a wide specificity towards substrates like glycosyl-*N*-acylsphingosines and flavonoid glycosides [26]. It is transport-competent *per se* and shares striking similarities with bacterial ß galactosidase. Domain IV contains the lactase active site and is anchored to the membrane via one single pass domain containing the lactase active site. Unlike domain III, this domain is not transport-competent *per se* and requires the presence of domain III to be trafficked along the secretory pathway (LPHßfinal) [24].

**Figure 1:**
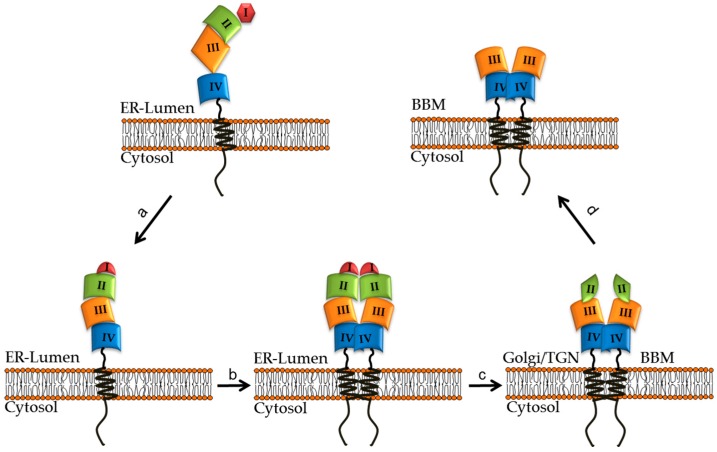
Folding and maturation steps of intestinal lactase-phlorizin hydrolase (LPH) along the secretory pathway to the brush border membrane (BBM). (**a**) The synthesized monomeric pro-LPH is translocated in the endoplasmic reticulum (ER) where it acquires proper folding via binding to calnexin. (**b**) The folded pro-LPH homodimerize before leaving the ER. (**c**) In the Golgi apparatus, the pro-LPH is glycosylated and proteolytically cleaved to LPHßinitial. (**d**) LPHßinitial is sorted to the apical membrane (BBM) where pancreatic trypsin cleaves LPHßinitial to its final mature LPHßfinal form (see references [22,25,30,31,35]).

LPH is exclusively expressed in the enterocytes, and its processing comprises three main steps that implicate the concerted interaction of the different domains [27]. The first crucial step in the life cycle of LPH is the acquisition of correct folding of a pro-LPH monomer that implicates the profragment that acts as an intramolecular chaperone and the small polypeptide L735-R868, referred to as a stretch that contains a binding site in and the ER-located chaperone calnexin [28]. A third equally important domain is the transmembrane domain that plays a key role in the homodimerization of pro-LPH, a step that constitutes a prerequisite for the ER exit and acquisition of enzymatic function. In fact, the removal of this domain results in a monomeric protein that is blocked in the ER as an immature and enzymatically-inactive mannose-rich polypeptide [29]. The second step along the secretory pathway of pro-LPH is its complex glycosylation and proteolytic cleavage in the Golgi [10]. The cleavage event occurs first at R734-L735 and releases an LPH molecule referred to as LPHßinitial that is targeted with high fidelity to the apical membrane [28,30]. In the apical membrane, the polypeptide stretch L735-R868 is released from the final LPH form (LPHßfinal) by luminal trypsin and the brush border LPH protein is generated that comprises the functional domains III (phlorizin hydrolase activity) and domain IV (lactase activity) [31,32] (Figure 1). The high level of *N*- and *O*-glycosylation of LPH is involved in the regulation of the trafficking efficiency to the apical membrane and the enzymatic capacity [33,34]. In fact, an *N*- and *O*-glycosylated LPH is almost 4 fold more active than the *N*-glycosylated enzyme [34]. Moreover, reduced glycosylation leads to less exposure of LPH at the cell surface.

## 5. Current Knowledge of CLD Mutations

**Figure 2:**
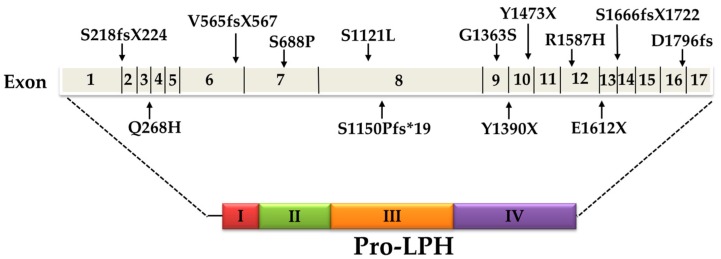
Schematic presentation of mutations in congenital lactase deficiency (CLD) and their location in the exons of the gene of lactase-phlorizin hydrolase (LPH). I-IV correspond to the four homologous domains of pro-LPH (see references [17,23,36,37]).

The pattern of inheritance of CLD can be both homozygote and compound heterozygote. Several different mutations in the coding region of LPH have been meanwhile characterized, the majority of them in a study with 32 Finish patients. Around 80% of the patients were homozygous for a stop codon at tyrosine 1390 (Y1390X) that results in a truncated protein. The presence of this mutation, referred to as the Fin (major) mutation, was explored in the LPH gene in a cohort of more than 550 individuals in Finland and revealed frequencies varying from 1:35 to 1:133. Other mutations, S1666fsX1722 and S218fsX224, resulted in a frameshift and a premature stop codon or an amino acid substitution (Q268H and G1363S). The Fin (major) Y1390X mutation was found in two more patients of Finnish origin in a compound heterozygote pattern of inheritance with either R1587H or V565fsX567 [17]. Two more mutations were identified in an Italian patient, one resulting in an amino acid substitution S688P and the other in a stop codon E1612X. These two mutations were not found in a cohort of about 100 Italian control individuals not suffering from lactose malabsorption indicating that the mutations are rare [36]. The only mutation common to the Finnish population and other ethnic groups is the G1363S, which was also found in a homozygous state in two Turkish siblings [17,36] and in an Iraqi patient (R. Santer, University Clinic Eppendorf, Hamburg, personal communication). Another mutation in a Turkish patient predicted a frameshift (S1150Pfs*19) and a premature termination of translation pro-LPH [37]. Finally, deletion mutations were also detected in a Japanese infant that led to a termination of the translation (Y1473X) and to a frameshift (D1796fs) in domain IV of pro-LPH [38,39] (Figure 2, Table 1).

## 6. Impact of CLD Mutations on the Structural and Biosynthetic Features of LPH

The sophisticated interaction between the different domains of pro-LPH, each with its specific role in the context of folding, trafficking competence, polarized sorting, and enzymatic function of pro-LPH could be destabilized or severely altered by mutations in either one of these domains. Until present, only two published studies have analyzed the genotype-phenotype relationship in CLD at the molecular and cellular levels. One of these studies focused on the impact of the G1363S mutation, which was characterized in Finnish, Turkish, and Iraqi patients and is localized in domain III and in close proximity downstream the phlorizin hydrolase active site. This mutation elicited misfolding, not only in domain III but also of entire pro-LPH resulting in a transport-incompetent LPH-G1363S that was intracellularly blocked in the ER. Given that domain III is an autonomous and transport-competent *per se*, these findings highlight the central role of this domain in the overall folding pro-LPH. Remarkably, the G1363S creates an additional *N*-glycosylation site and confers a temperature-sensitive phenotype on the LPH-G1363S mutant, which acquires transport-competence and can exit the ER at a temperature of 20°C. These characteristics suggest that a longer exposure of this mutant to the folding machinery in the ER, for instance to the chaperones calnexin and BiP, may help restore the correct folding of the LPH domains. Chaperone-based therapies can thus be a promising strategy in the treatment of CLD patients carrying this mutation [40].

Another study focused on the biochemical features of two mutations, Y1473X and D1796fs, which were found as a compound heterozygous pattern in a Japanese infant with CLD [39]. These mutations are in domain IV of LPH and result either in partial truncations of homologous domain IV and the entire transmembrane domain and the cytosolic tail. Both LPH mutants were retained in the ER as enzymatically-inactive proteins, despite the presence of the lactase (Glu1749) and the phlorizin hydrolase (Glu1273) sites in the frameshift mutant LPH-D1796fs, and the phlorizin-hydrolase at Glu1273 in the LPH-Y1473X. It is obvious, therefore, that the compound heterozygote pattern of two misfolded and functionally inactive mutants is responsible for the absence of enzymatic activity and subsequently lactose intolerance in the infant [38].

Although detailed analyses of the other known mutation in CLD do not exist, the pathogenic severity can be predicted if a mutation has generated a truncated protein or if a mutation is located in a region that is critically important in the context of folding and acquisition of transport competence. For instance, the mutations Y1390X and E1612X generate pro-LPH mutants that lack the critical domains IV and also the transmembrane domain, which plays a primordial role in the homodimerization of pro-LPH [36,38]. Since this event constitutes an absolute requirement for pro-LPH to exit the ER, transmembrane truncation will certainly end with a pro-LPH protein that is retained in the ER and has lost its function. A similar scenario can also be proposed for the frameshift mutations S1150Pfs*19 and S1666fsX1722. The S218fsX224 generates a protein that contains only a short polypeptide in domain I and none of the catalytic sites are prone to be immediately degraded [17].

The mutations Q268H and S688P are located in the profragment of pro-LPH that exerts a central role as an intramolecular chaperone in the initial folding of pro-LPH prior to homodimerization. The S688P was predicted to be possibly damaged by PolyPhen, and the substitution of a basic histidine for uncharged glutamine in Q268H was likely to alter the secondary structure of the profragment [28,35]. Finally, the R1587H that is located in domain IV was also predicted to be possibly damaged by PolyPhen.

Obviously, the majority of the mutations, if not all, that have been so far characterized in CLD, whether in a homozygous or a compound heterozygous pattern of inheritance can be considered to be pathogenic, unlike mutations of SI in CSID that have been categorized into three major classes according to their trafficking behavior and grade of functional severity [28,35].

## 7. Are CLD Mutations Symptomatic in Heterozygote Carriers?

Until present, it is unknown whether a single normal parental allele in conjunction with the diseased one would be enough to generate and process LPH that can sufficiently digest dietary lactose and would provide knowledge on the lactose digestive capacity of heterozygote carriers of severe pathogenic mutations in the LPH gene. The question that should be addressed in this context is whether an interaction between a wild type LPH monomer with another LPH mutant monomer resulting in a heterodimer is potentially possible. This assumption stems from the fact that wild type LPH dimerizes in the ER to an active and transport-competent enzyme. One of the absolute requirements for this early event along the secretory pathway is the existence of an intact, that is, non-mutated transmembrane domain. It can be, therefore, hypothesized that an interaction between wild type LPH and LPH mutants containing transmembrane domains, such as LPH-G1363S, LPH-Q268H, LPH-S688P, and LPH-R1587H, may take place. Further, the trafficking and enzymatic function of the resulting heterodimers would be dictated by the severity of the mutation itself. It would be tempting, therefore, to address this question in future co-expression studies of wild type LPH and its transmembrane domains carrying mutants. Obviously, this type of interaction excludes the truncated mutants that lack the transmembrane domains, such as LPH-Y1390X, LPH-E1612X, LPH-D1796fs, LPH-Y1473X, LPH-S1150Pfs*19, LPH-S1666fsX1722, and LPH-S218fsX224. Nevertheless, interaction via the luminal region of these mutants rather than the transmembrane anchor cannot be excluded, provided minimal folding requirements are fulfilled, such as correct folding of domains involved in dimerization of LPH. In a recent study, this hypothesis has been examined for two truncated LPH mutants, LPH-D1796fs, LPH-Y1473X, in a Japanese infant. In co-expression studies, neither the LPH-Y1473X nor LPH-D1796fs were retained in the same experimental sample as wild type LPH clearly indicating that these mutant forms do not interact with wild type LPH [38]. However, these mutants do not assume correct folding and, therefore, are not expected to interact with a luminal domain of wild type LPH.

## 8. Future Perspectives

The current progress made in the identification of several mutations in CLD is certainly essential at the clinical level, facilitates the genetic testing, and moreover shows that this disease is more frequent than previously thought. Nevertheless, this progress is not paralleled by functional and biochemical analysis of the LPH mutants. In particular, the analysis of the effect of missense mutations on the folding pattern, function, and pathogenicity of LPH mutants determine the grade of severity of the protein phenotype. These studies are also crucial for the heterozygote in light of the potential interaction of wild type and mutant LPH via the transmembrane domain, resulting in the effect on the overall functional performance and trafficking competence of a potential heterodimeric LPH. Furthermore, despite the view that a truncated protein is a priori not active or is transport-competent, correct folding or partial folding of the extracellular domain may still have the capability to form dimers with wild type LPH and should be studied. Until solid data on the effect of these mutations on the heterozygotes are generated and given the severity of this disease and life threat in the early days for the newborn, genetic testing of the LPH gene is recommended for potential heterozygote carriers.

## Figures and Tables

**Table 1:** Mutations in CLD, their location in the LPH gene, inheritance pattern, and effects. CLD: congenital lactase deficiency; LPH: lactase-phlorizin hydrolase.

LPH-Mutation		Location	Mutation Effect	Inheritance Pattern (Genotype)	Ethnic Origin	Reference
cDNA	Protein					
c.4170T > A	p. Y1390X	Exon 9	Nonsense (truncating)	Homozygote	Finland	[1]
c.4998_5001delTGAG	p. S1666fsX1722	Exon 14	Frameshift (truncating)	Compound heterozygote		
c.653_654delCT	p. S218fsX224	Exon 2	Missense	Compound heterozygote		
c.804G > C	p. Q268H	Exon 3	Missense	Compound heterozygote		
c.4087G > A	p. G1363S	Exon 9	Missense	Compound heterozygote		
c.1692-1696delAGTGG	p. V565fsX567	Exon 6	Frameshift (truncating)	Compound heterozygote		
c.4760G >A	p. R1587H	Exon 12	Missense	Compound heterozygote		
c.2062T > C	p. S688P	Exon 7	Missense	Compound heterozygote	Italy	[2]
c.4834G > T	p. E1612X	Exon 12	Nonsense (truncating)			
c.4419C > G	p. Y1473X	Exon 10	Nonsense (truncating)	Compound heterozygote	Japan	[3]
c.5387delA	p. D1796fs	Exon 16	Frameshift (truncating)			
c.4087G > A	p. G1363S	Exon 9	Missense	Homozygote	Turkey	[2]
c.3448delT	p.S1150Pfs*19	Exon 8	Frameshift (truncating)	Homozygote		[4]
c.4087G > A	p. G1363S	Exon 9	Missense	Homozygote	Iraq	Not published
c.3362C > T	p. S1121L	Exon 8	-	Homozygote	-	Not published
